# MALDI-TOF/MS Serum Spectral Profiling for Breast Cancer Discrimination Using Direct and ACN-Assisted Workflows: A Retrospective Proof-of-Concept Study

**DOI:** 10.3390/diagnostics16213520

**Published:** 2026-10-27

**Authors:** Donggun Seo, Soojeong Choi, Hyeongjin Kweon, Sae Byul Lee, Dong-Chan Kim

**Affiliations:** 1 R&D Center, ASTA Inc., 767, Sinsu-ro, Yongin-si 16827, Republic of Korea; seodonggun3163@naver.com (D.S.); hyeongjin.kweon@astams.com (H.K.); 2 Department of Surgery, Seoul Medical Center, 156, Sinnae-ro, Jungnang-gu, Seoul 02053, Republic of Korea; ssujevi@naver.com; 3 Division of Breast Surgery, Department of Surgery, Asan Medical Center, University of Ulsan College of Medicine, 88, Olympic-ro 43-gil, Songpa-gu, Seoul 05505, Republic of Korea

**Keywords:** matrix-assisted laser desorption/ionization time-of-flight mass spectrometry, breast cancer, serum proteomics, serum peptidomics, acetonitrile precipitation, machine learning, batch effect, proof-of-concept

## Abstract

**Background/Objectives:** Serum matrix-assisted laser desorption/ionization time-of-flight mass spectrometry (MALDI-TOF/MS) profiling may complement breast cancer (BrC) assessment, but retrospective studies are vulnerable to demographic and batch confounding. We evaluated direct-serum (Workflow A) and acetonitrile (ACN)-assisted (Workflow B) profiling. **Methods:** Workflow A analyzed 60 archived sera (30 BrC, 30 controls) with sample-level nested 10-fold cross-validation and L2-regularized logistic regression. Workflow B included 40 sample groups (20 BrC, 20 negative/comparator; approximately four spots/sample); replicates were aggregated before repeated sample-level five-fold cross-validation and batch/source diagnostics. **Results:** Workflow A yielded an area under the receiver operating characteristic curve (AUC) of 0.932, but groups were not age matched (52 vs. 45 years; standardized mean difference approximately 1.03), precluding cancer-specific interpretation independent of age. Workflow B yielded a mean AUC of 0.978 ± 0.007 and a balanced accuracy of 0.968, but negative/comparator batch/date classification reached an AUC of 0.970, and cross-batch transfer was asymmetric (1.00 vs. 0.64); five comparator sera were commercially sourced. **Conclusions:** Workflow A provides preliminary group-discrimination evidence but remains age-confounded. Workflow B demonstrates strong apparent internal discrimination under substantial batch/source confounding and is best viewed as an analytical stress test, not independent validation. Candidate m/z signals remain unidentified; age-matched, batch-balanced external validation and orthogonal molecular identification are required before cancer-specific or biomarker claims.

## 1. Introduction

Breast cancer (BrC) remains one of the most frequently diagnosed malignancies among women worldwide. Imaging-based screening and diagnostic modalities, including mammography, ultrasonography, and magnetic resonance imaging, remain central to detection and clinical management, but interest continues in developing complementary minimally invasive blood-based approaches that could support triage, risk stratification, or longitudinal assessment [1,2,3,4,5,6,7].

Matrix-assisted laser desorption/ionization time-of-flight mass spectrometry (MALDI-TOF/MS) can generate complex proteomic and peptidomic fingerprints rapidly from biological fluids. Beyond its established use in clinical microbiology, MALDI-TOF/MS has been explored for serum- and plasma-based cancer profiling [8,9,10,11,12,13]. Previous BrC studies have reported discriminatory serum protein or peptide patterns using MALDI- or surface-enhanced laser desorption/ionization-based approaches, including case-control and independent validation designs [14,15,16]. These studies support the biological plausibility of serum spectral classification while also emphasizing the sensitivity of serum peptidomics to sample preparation, storage, processing order, and analytical batch [17,18].

Serum N-glycan profiling has also been investigated for BrC discrimination. Lee et al. previously demonstrated BrC classification using serum N-glycan MALDI-TOF/MS analysis [19]. Accordingly, the present work does not position glycan profiling as a new contribution. Instead, the primary methodological question is whether simplified protein/peptide-oriented serum preparation can preserve disease-associated discriminatory information without enzymatic glycan release or extensive fractionation.

Acetonitrile (ACN) precipitation provides one simple approach for reducing abundant high-molecular-weight serum proteins while retaining a low-molecular-weight fraction for mass-spectrometric analysis. Organic-solvent extraction and ACN enrichment of low-molecular-weight serum proteins have been described previously [20,21], and ACN-supernatant MALDI peptidomic workflows have been applied in biomarker research [22]. Such simplicity may be attractive for translational workflows, although it also makes strict control of pre-analytical and plate/run conditions essential.

Machine-learning methods can assist with the classification of high-dimensional spectra, but methodological rigor is critical. Technical replicates must not be treated as independent samples, feature selection must be restricted to training data, and performance estimates should be interpreted alongside batch diagnostics and independent validation [17]. A high area under the receiver operating characteristic curve (AUC) alone does not establish disease specificity if the disease labels are correlated with the collection source, measurement date, plate, or processing order.

We evaluated two serum spectral workflows, but they are not treated as biological replicates of one another. Workflow A is the primary direct-serum proof-of-concept analysis. Workflow B is a separate ACN-assisted exploratory analytical experiment used to examine how apparent classification performance behaves in the presence of source and acquisition-batch heterogeneity. The workflows involved different sample sets, preparation methods, m/z representations, and acquisition structures; therefore, similar AUC values do not establish that the same biological signals were detected. The archived materials available for this revision did not provide a unified cross-workflow sample-identifier map sufficient to verify sample overlap; so, no claim of biological independence or cross-workflow replication is made.

## 2. Materials and Methods

### 2.1. Study Overview and Analytical Workflows

This retrospective proof-of-concept study evaluated two MALDI-TOF/MS serum profiling workflows in parallel but interpreted them separately. Workflow A was the previously defined direct serum protein/peptide analysis in a 60-sample subset (30 BrC and 30 controls) and provides the primary proof-of-concept evidence. Workflow B was an ACN-assisted exploratory experiment comprising 40 sample groups (20 BrC and 20 negative/comparator samples) analyzed as approximately four technical spots per sample. Workflow B is used primarily as an analytical stress test of replicate handling, batch/source structure, and apparent discrimination under a second preparation strategy. Performance from the two workflows was not pooled, statistically compared, or interpreted as evidence that the same biological feature set was reproduced (Table 1).

### 2.2. Source Cohort, Ethics, and Direct-Profiling Study Set

The clinical serum samples used in both Workflow A and Workflow B were obtained from female participants treated at Asan Medical Center under Institutional Review Board approval (Receipt No.: S2021-0995-0001/Project No.: 2021-0672). Written informed consent was obtained in accordance with institutional and ethical guidelines. The source clinical collection initially comprised 573 serum samples, including 317 BrC cases and 256 controls. Following the quality-control (QC) and preprocessing exclusions used for the source study, 562 samples remained (306 BrC and 256 controls).

The direct-profiling proof-of-concept analysis used a randomly selected 60-sample subset comprising 30 BrC cases and 30 controls. Cases had histologically confirmed BrC. Controls had no history of cancer and were classified as Breast Imaging Reporting and Data System (BI-RADS) I–III. The groups were not age matched: the BrC group had a mean age of 52 years (SD 7.7) and controls 45 years (SD 5.8), corresponding to a descriptive standardized mean difference of approximately 1.03 based on the reported summary statistics. No prespecified age-adjusted classifier was part of the archived analysis; therefore, the reported Workflow A AUC cannot be interpreted as cancer-specific independently of age. This demographic imbalance is treated as a major unresolved confounder throughout the revised interpretation.

### 2.3. Direct Serum Protein/Peptide Preparation and MALDI-TOF/MS Acquisition

For Workflow A, serum was diluted 1:5 with deionized water. Five microliters of diluted serum were mixed with 10 µL of matrix solution and prepared for target spotting. Mass spectra were acquired using the IDSys LT^®^ MALDI-TOF mass spectrometer (ASTA Inc., Suwon-si, Republic of Korea) in linear positive-ion mode over m/z 2000–30,000. For each sample, 1.5 µL of the sample-matrix mixture was spotted in quadruplicate onto a 96-well stainless-steel target. The matrix consisted of 40 mg/mL sinapinic acid in 70% ACN with 0.1% trifluoroacetic acid. Each spot received 1200 laser shots (40 shots at 30 discrete loci).

QC criteria included signal-to-noise ratio > 3, absence of abnormal baseline distortion, and consistent internal-calibration peak detection. Spectra failing these criteria were excluded. Technical replicates were averaged before downstream modeling so that each biological serum sample contributed one sample-level observation.

### 2.4. ACN-Assisted Serum Preparation and Experimental Set

The archived experimental plan documented the Workflow B preparation and acquisition procedures summarized below. The experiment included 20 BrC sera supplied by Asan Medical Center, 15 non-breast (Non-Br) clinical sera supplied by Asan Medical Center, and 5 commercially obtained Lampire sera (Cat. No. 7323801) used as additional negative/comparator material. The available records identify 40 sample groups and 160 spectra, corresponding numerically to four technical spots per sample. The five Lampire sera were not part of the Asan Medical Center clinical cohort, and donor age, collection conditions, storage history, and other donor-level metadata were unavailable. Because clinical and commercial materials were combined, biological and pre-analytical comparability of the negative/comparator group cannot be assumed. Accordingly, Workflow B is not treated as a clinical validation cohort.

For ACN preparation, 20 µL of 100% HPLC-grade ACN (Supelco; Cat. No. 1.00030) was dispensed into a 0.5 mL tube, followed by 10 µL of serum (ACN:serum = 2:1, *v*/*v*). The mixture was mixed and centrifuged at 12,000 rpm for 5 min. Avoiding the precipitated pellet, 1.5 µL of supernatant was spotted in four positions on a 96-well MALDI plate and dried. Matrix (1.5 µL per spot) was then added and dried. ACN and matrix negative-control spots were prepared in parallel, and spectra were acquired on an IDSys LT^®^ instrument (instrument unit no. 49). The archived plan documented the instrument identity, ACN reagent, sample and ACN volumes, centrifugation, four technical spots per sample, 96-well plate use, drying, matrix addition volume, and ACN/matrix negative-control spots. It did not document the matrix formulation or concentration, laser power/energy, laser-shot count, calibration material or calibration procedure, acquisition polarity, plate-level pooled QC, serum storage duration, or number of prior freeze–thaw cycles. These missing parameters were not reconstructed or inferred retrospectively. The analyzed dataset covered m/z 1000–19,500 at 5 m/z intervals.

The BrC samples in the ACN set had a mean age of 48.25 years (range 31–66). The 15 clinical Non-Br samples had a mean age of 50.8 years (range 40–62); age was unavailable for the five Lampire samples. The acquisition structure was not batch balanced: BrC samples were measured on 7 August 2026 on plates P1 and P2, whereas negative/comparator samples were distributed across 7 August 2026 plate P3 and 12 August 2026 plate P2. Thus, the disease status, specimen source, date, and plate were partially entangled by design, precluding separation of cancer-associated effects from analytical/source effects within Workflow B alone.

### 2.5. Spectral Preprocessing and Technical-Replicate Handling

For Workflow A, raw spectra were processed using IDSys LT^®^ proprietary software with baseline subtraction, Savitzky–Golay smoothing, internal calibration, peak detection at signal-to-noise ratio > 3, and m/z alignment. Total ion current (TIC) normalization was applied before statistical analysis.

For Workflow B, the analysis dataset contained 3701 peak-height features covering m/z 1000–19,500 at 5 m/z intervals. Spectrum-level TIC normalization was followed by log1p transformation. Technical replicates from the same sample ID were not treated as independent observations: replicate spectra were aggregated at the sample level using the mean for the primary analysis, with median aggregation examined as a sensitivity analysis. This sample-level handling prevented spectra from the same biological/sample group from being split across training and test folds. Inter-day/inter-plate reproducibility was not prospectively assessed using a shared pooled-QC specimen across all acquisition structures; therefore, batch diagnostics were interpreted as post hoc evidence of analytical confounding rather than as a formal QC correction procedure.

### 2.6. Feature Selection, Classification, and Cross-Validation

Workflow A used sample-level nested stratified 10-fold cross-validation. Feature selection was conducted independently within each outer-training fold using one-way analysis of variance with Benjamini–Hochberg false-discovery-rate correction. An L2-regularized logistic regression model was trained on selected features. Regularization strength was tuned only within the training data, and performance was evaluated on held-out outer folds.

For Workflow B, classification used regularized logistic regression under repeated sample-level five-fold cross-validation (10 repeats). Feature selection was restricted to the training fold for each split; no disease-associated feature was globally selected before hold-out evaluation. AUC and balanced accuracy were summarized across repeats. The analysis also calculated selection frequency across repeated folds as a descriptive measure of candidate m/z stability. Univariate m/z AUC values and effect sizes were used only for exploratory candidate ranking and were not interpreted as independent validation metrics.

### 2.7. Batch-Effect and Technical-QC Analyses for the ACN Workflow

Because disease labels were partially associated with measurement date and plate in Workflow B, batch diagnostics were specified separately from disease classification. First, a classifier was used to distinguish the two negative/comparator acquisition structures (7 August P3 vs. 12 August P2). Second, plate discrimination among same-day BrC samples (P1 vs. P2 on 7 August) was assessed. Third, cross-batch transfer was evaluated in both directions by training on one disease/batch configuration and testing on the alternate configuration. Principal component analysis (PCA) coordinates were visualized both by disease label and measurement batch to assess whether disease and batch structure coexisted in the same feature space.

Technical-replicate reproducibility was assessed by within-sample spectral correlation. The analysis report identified generally high replicate similarity but also a clear outlier sample (BrC sample 6146), which motivated sensitivity analysis using median rather than mean replicate aggregation. No technical replicate was counted as an independent patient or independent biological sample.

### 2.8. Statistical Interpretation

The AUC was treated as the principal discrimination metric, but the AUC was not interpreted as evidence of disease specificity when important confounding remained. For Workflow A, the substantial age imbalance is an unresolved demographic confounder. For Workflow B, disease status was partially associated with date, plate, and specimen source. The mean AUC across the 10 repeated five-fold evaluations was therefore retained only as an internal apparent-discrimination estimate. The AUC obtained by averaging repeated out-of-fold predictions for each sample was treated as secondary and descriptive, because repeated predictions from the same samples are not equivalent to external validation. Candidate m/z values are reported as unidentified discriminatory m/z bins, not molecularly identified biomarkers, and no molecular concordance between Workflows A and B is claimed.

## 3. Results

### 3.1. Workflow A: Direct Serum Profiling

The direct-profiling analysis included 30 BrC and 30 control serum samples. All participants were female. BrC cases in the available clinical subset were predominantly stage II–III, whereas controls were BI-RADS I–III. The BrC group had a mean age of 52 years (SD 7.7; range 34–64), compared with 45 years (SD 5.8; range 36–56) in the controls (Table 2). This 7-year mean difference corresponds to a descriptive standardized mean difference of approximately 1.03, indicating substantial demographic imbalance. For descriptive context, under a simple equal-variance Gaussian approximation, the standardized mean difference of 1.03 corresponds to an age-only separation AUC of approximately 0.77 [AUC = Φ(d/√2)]; this is a summary-statistic approximation, not a fitted or validated age classifier. Consequently, age-related serum spectral differences may contribute to the observed separation, and Workflow A cannot establish cancer-specific discrimination independent of age.

**Figure 1:**
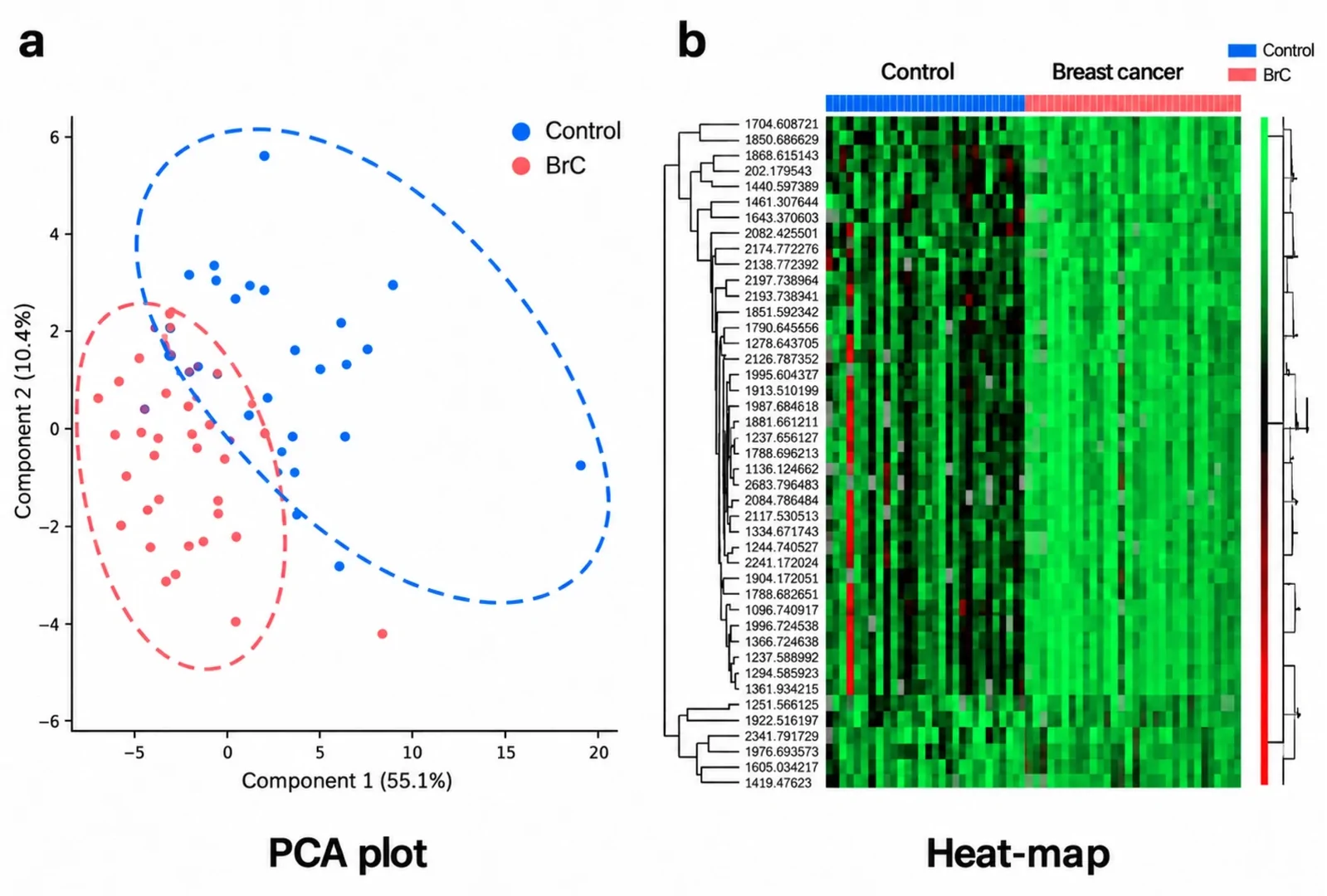
Direct serum MALDI-TOF/MS profile comparison in the 60-sample subset. (**a**) Principal component analysis of 30 BrC and 30 control serum samples. (**b**) Heatmap displaying differential clustering of selected m/z signals. The unsupervised plots illustrate group-level structure but are not diagnostic-performance estimates. PCA, principal component analysis; BrC, breast cancer; MALDI-TOF/MS, matrix-assisted laser desorption/ionization time-of-flight mass spectrometry.

PCA and hierarchical clustering demonstrated group-level spectral differences with partial overlap (Figure 1 and Figures S1–S3). These unsupervised analyses were used for visualization only and were not used to derive the AUC.

**Figure 2:**
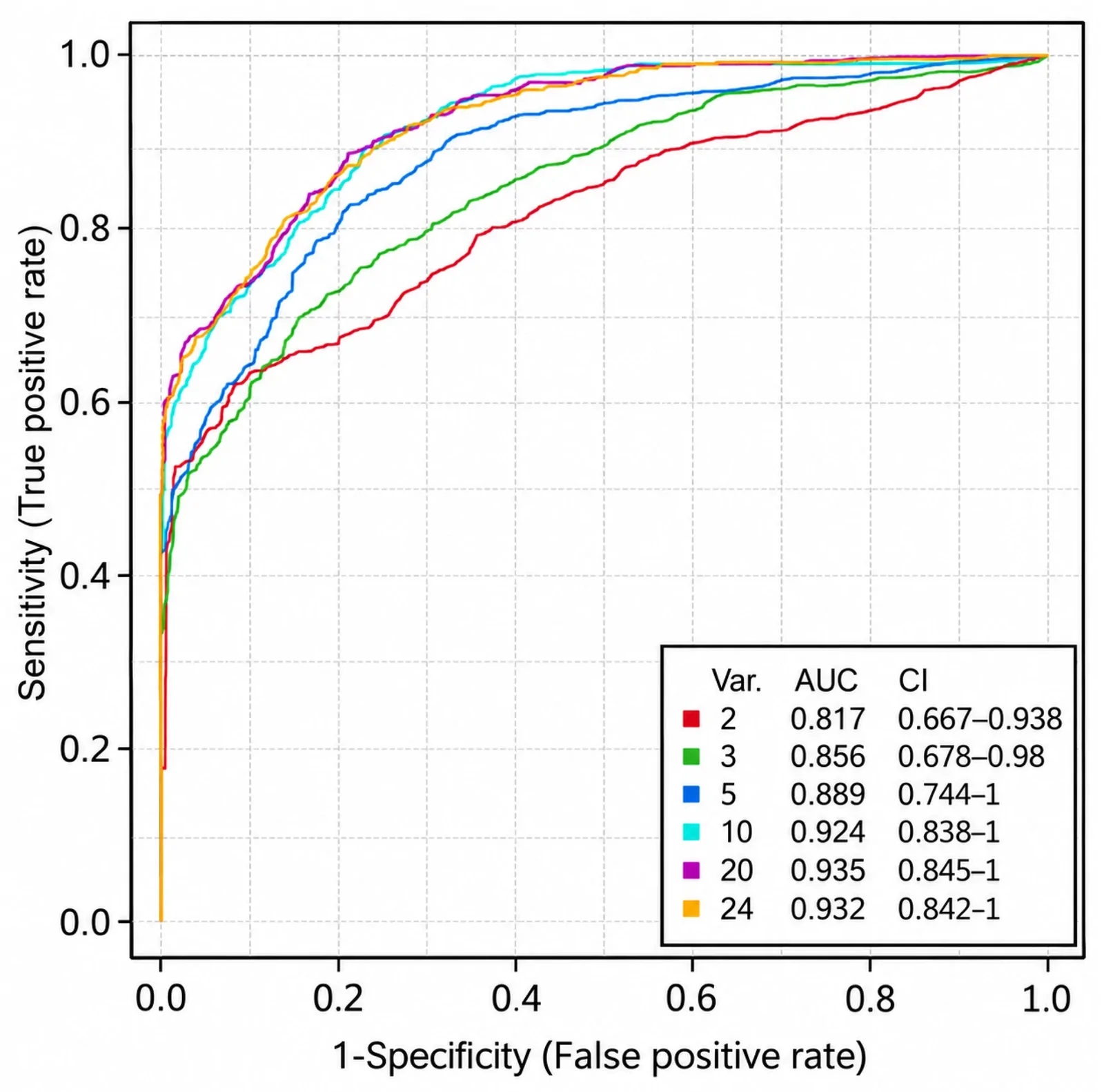
Receiver operating characteristic curves for direct serum protein/peptide classification using different numbers of selected m/z features. The 24-feature model yielded an AUC of 0.932; the confidence interval shown in the source figure was 0.842–1.000. Performance derives from the retrospective 60-sample subset and is not external validation. AUC, area under the receiver operating characteristic curve; CI, confidence interval.

Using sample-level nested cross-validation with training-fold-only feature selection, the L2-regularized logistic-regression model yielded an AUC of 0.932. The ROC analysis across feature-count settings is shown in Figure 2. Representative direct-workflow spectrum comparisons are shown in Figure S4. Because the source analysis did not provide a single unambiguous pooled confusion matrix for the 60 samples, the sensitivity, specificity, and accuracy values were not carried forward as headline results. In addition, because age was not matched, and no prespecified age-adjusted model was validated, the AUC should be interpreted as overall group discrimination rather than cancer-specific performance.

### 3.2. Workflow B: ACN-Assisted Experimental Set

The ACN-assisted experiment comprised 20 BrC and 20 negative/comparator sample groups, generating 160 spectra in total. The comparator material consisted of 15 clinical Non-Br sera from Asan Medical Center and five commercially obtained Lampire sera. Technical spots were aggregated to the sample level before analysis, resulting in 40 analysis units rather than 160 independent observations (Table 3). Because clinical and commercial sera were mixed within the comparator class, and the commercial donor metadata were unavailable, the comparator group is heterogeneous in specimen source and cannot be regarded as a biologically uniform control population.

**Figure 3:**
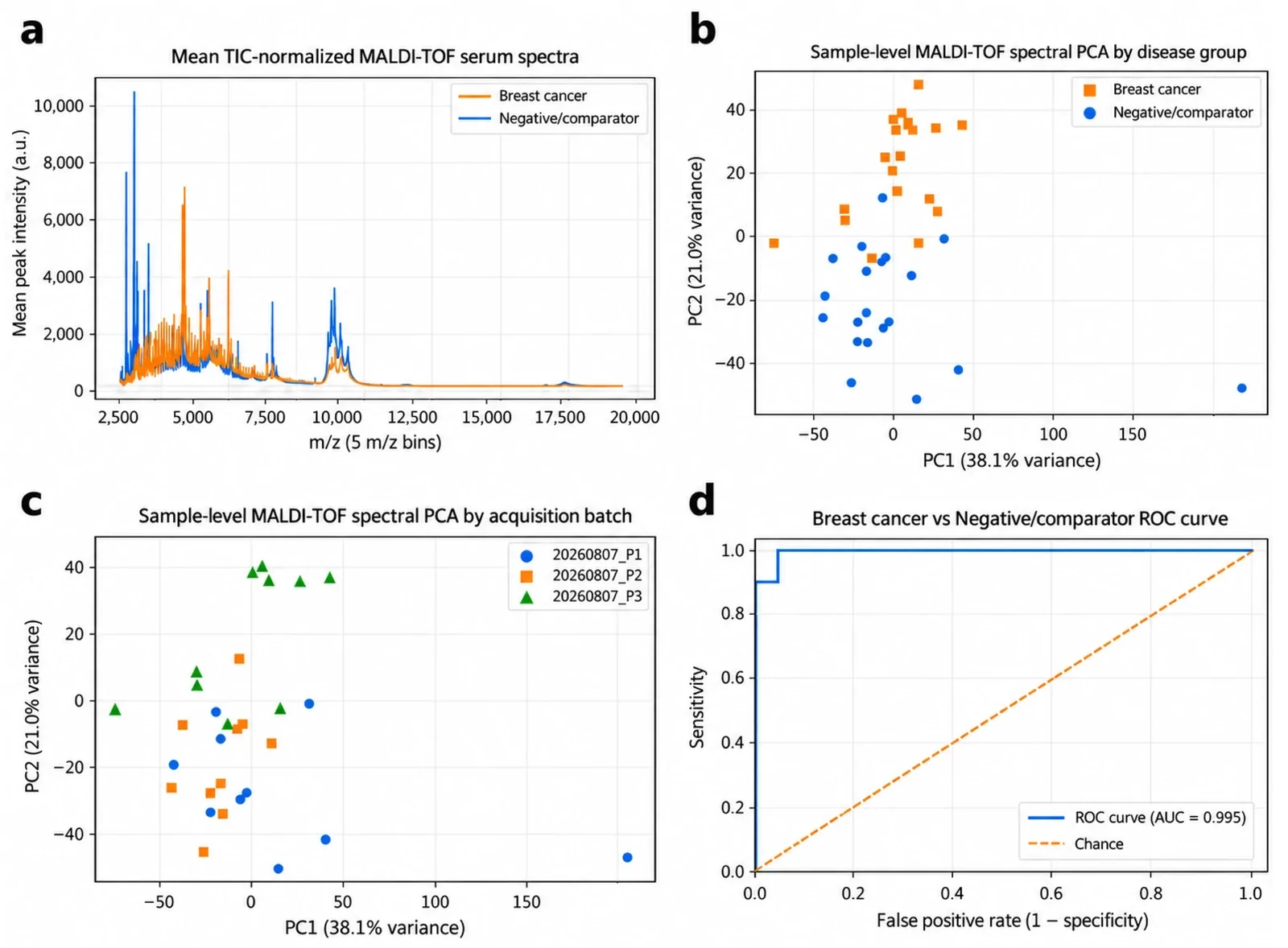
ACN-assisted serum MALDI-TOF/MS exploratory analysis. (**a**) Mean TIC-normalized MALDI-TOF serum spectra for breast cancer and negative/comparator samples. (**b**) Sample-level MALDI-TOF spectral PCA colored by disease group. (**c**) The same PCA representation colored by acquisition batch, demonstrating concurrent batch structure. (**d**) Breast cancer vs. negative/comparator ROC curve from repeated out-of-fold disease classification. The ACN experiment contained 40 sample groups with approximately four technical spots per sample; replicates were aggregated before modeling. ACN, acetonitrile; TIC, total ion current; MALDI-TOF/MS, matrix-assisted laser desorption/ionization time-of-flight mass spectrometry; PCA, principal component analysis; ROC, receiver operating characteristic curve.

The TIC-normalized mean spectra showed broad group differences across multiple m/z regions. PCA colored by disease label showed substantial structure; however, the same coordinates colored by acquisition batch also showed strong batch-related structure (Figure 3). Because the disease status, source, date, and plate were not orthogonally balanced, the apparent group separation cannot be confidently assigned to malignancy. Workflow B is therefore interpreted as an analytical stress test that illustrates the risk of confounded serum spectral classification.

### 3.3. Cross-Validated Discrimination in the ACN Workflow

Across 10 repeats of sample-level five-fold cross-validation with feature selection confined to the training folds, the mean AUC was 0.978 ± 0.007, and the mean balanced accuracy was 0.968; individual repeat AUCs ranged from 0.9675 to 0.9900 (Table S1). These values represent strong apparent internal discrimination, not evidence of cancer-specific classification, because the same dataset contains substantial date/plate/source structure. They are not external validation estimates.

A secondary analysis averaged the repeated out-of-fold prediction probabilities for each sample and produced an AUC of 0.995. At a probability threshold of 0.5, sensitivity was 1.00 and specificity was 0.95. Because each sample contributed predictions from multiple repeated cross-validation runs, this aggregated value was considered descriptive and was not used as the primary performance claim (Table 4).

### 3.4. Candidate Discriminatory m/z Bins and Replicate QC

The ACN analysis identified multiple m/z bins with large effect sizes and repeated selection across cross-validation splits. Among the highest-priority bins were m/z 5155, 1480, and 7305, with additional exploratory bins listed in Table S2 and Figure S6. These values are 5 m/z bins and have not been molecularly identified. Because Workflow A and Workflow B used different preparation methods, m/z representations, and feature-selection procedures, no exact cross-workflow molecular-feature concordance was established or claimed. The ACN features are therefore reported only as unidentified discriminatory spectral bins and not as peptides, proteins, adducts, biomarkers, or evidence that the same molecular signal was reproduced across workflows. As a descriptive cross-workflow check, the six signals highlighted in the direct workflow (Figure S4; m/z ≈ 2101, 2550, ~3258, 4150, 4207, and 5001) were compared with the ten prioritized ACN bins in Table S2. Only one near-coincident pair was observed (direct m/z 4150 vs. ACN bin 4155; Δ ≈ 5 Da, ≈0.12%). No ACN bin fell within ±5 Da of the other five direct-workflow signals, and the ACN bins at m/z 1220 and 1480 lie below the direct-workflow acquisition range. Given the different preparation methods and m/z representations, this single proximity is reported descriptively and is not interpreted as evidence of a shared molecular species or cross-workflow biological replication (Table S4).

Most samples showed high within-sample correlation across technical spots, with a reported median of the mean within-sample correlation of 0.987. BrC sample 6146 was a notable technical outlier (mean replicate correlation 0.192; minimum correlation −0.001). Median replicate aggregation retained a high discriminatory signal in sensitivity analysis, supporting the conclusion that the overall result was not driven exclusively by a single aberrant technical replicate. Replicate-QC details are shown in Figure S7.

### 3.5. Batch-Effect Diagnostics

Batch diagnostics materially changed the interpretation of the high ACN discrimination result. Within the negative/comparator group, measurement structure on 7 August P3 vs. 12 August P2 was highly classifiable (AUC 0.970), demonstrating a strong non-disease batch/date signature. In contrast, the same-day plate discrimination among BrC samples (P1 vs. P2) was lower (AUC 0.636) (Table S3).

Cross-batch transfer was asymmetric. Training on one disease/batch configuration and testing on the alternative configuration yielded an AUC of 1.00 in one direction but only 0.64 in the reverse direction; the reverse-direction AUC improved to 0.74 when Lampire samples were excluded. The change after excluding the commercial sera demonstrates sensitivity to the specimen source, while the persistent instability indicates that batch/source effects remain substantial. Accordingly, Workflow B cannot support a claim of independent biological replication, batch-independent generalizability, or successful cancer classification.

## 4. Discussion

This revised proof-of-concept study does not treat the two workflows as concordant biological replications. Workflow A remains the primary direct-serum proof-of-concept analysis and yielded a cross-validated AUC of 0.932 in 60 samples. Workflow B produced a high internal AUC under a different ACN-assisted preparation, but its disease labels were substantially entangled with the date, plate, and specimen source. Accordingly, Workflow B is interpreted primarily as an analytical stress test showing how strong apparent discrimination can coexist with severe non-disease structure. Similar AUC values across the two workflows do not establish that the same biological signal was detected.

Workflow A also has an important demographic limitation. The BrC group was on average 7 years older than the control group (52 vs. 45 years; descriptive standardized mean difference approximately 1.03). Serum proteomic and peptidomic profiles may vary with age and other host factors; so, the possibility that part of the AUC 0.932 reflects age-related biology rather than malignancy cannot be excluded. No prespecified age-adjusted classifier was validated in this proof-of-concept analysis; therefore, Workflow A should be interpreted as group discrimination in an age-unmatched retrospective subset, not as evidence of cancer-specific performance.

The ACN experiment remains informative methodologically, because technical replicates were correctly grouped at the sample level, and feature selection was confined to training data, reducing pseudoreplication and information leakage. However, these statistical safeguards cannot correct an experimental design in which the disease status, date, plate, and source are not independently balanced. The comparator class itself was heterogeneous: 15 clinical Non-Br sera were combined with five commercial Lampire sera for which donor age and pre-analytical metadata were unavailable. The reverse cross-batch AUC changed from 0.64 to 0.74 when Lampire samples were excluded, reinforcing that the specimen source contributed materially to the feature space.

The reproducibility of Workflow B is also limited by incomplete archived acquisition metadata. The retained experimental plan documented the IDSys LT instrument identity, ACN reagent and volumes, centrifugation, four technical spots per sample, matrix-addition volume, 96-well plate use, and ACN/matrix negative controls. It did not record the matrix formulation/concentration, laser power or shot count, calibration material/procedure, acquisition polarity, serum storage duration, prior freeze–thaw history, or shared pooled-QC measurements across plates/dates. We deliberately did not infer or reconstruct these missing parameters. The reported ACN results should therefore be regarded as a retrospective exploratory analysis with limited procedural reproducibility.

The discriminatory m/z signals in Workflow B are hypothesis-generating only. They are 5 m/z bins rather than molecularly identified species, and the current study does not establish whether they represent peptides, proteins, fragments, adducts, or other ions. The two workflows also used different preprocessing and m/z representations; so, no exact molecular-feature overlap is claimed between them. Orthogonal identification by high-resolution tandem mass spectrometry or liquid chromatography–tandem mass spectrometry would strengthen biological interpretation, but such additional wet-laboratory identification was not feasible within this retrospective revision. We therefore consistently use the terms “unidentified discriminatory m/z feature/bin” and reserve biomarker claims for future orthogonally validated studies. A direct descriptive comparison was nevertheless performed: among the six Figure S4 signals and the ten prioritized ACN bins, only m/z 4150 and the 4155 bin were near-coincident (Δ ≈ 5 Da, ≈0.12%). The other direct-workflow signals had no ACN bin within ±5 Da, while ACN bins 1220 and 1480 were outside the direct-workflow acquisition range. This limited proximity does not establish shared molecular identity or biological replication.

The direct workflow nevertheless remains the more interpretable of the two analyses, because its acquisition structure is not known to have the same severe date/plate confounding identified in Workflow B. Its AUC of 0.932 is therefore retained as the principal proof-of-concept result. However, the direct set is small, retrospective, age-unmatched, single-center, and includes BI-RADS II–III controls. These limitations preclude claims of validated clinical performance or disease specificity.

The principal limitations are as follows: (1) substantial age imbalance in Workflow A; (2) absence of a prospectively enrolled external validation cohort; (3) mixing of 15 clinical and five commercial comparator sera in Workflow B; (4) entanglement of disease status with acquisition date/plate/source in Workflow B; (5) incomplete archived acquisition, storage, calibration, and cross-plate QC metadata for Workflow B; (6) high dimensionality relative to the 40 Workflow B sample groups; (7) lack of molecular identification of candidate m/z bins; and (8) inability to verify the cross-workflow sample overlap from a unified archived identifier table. These limitations are central to interpretation rather than secondary caveats.

Future validation should therefore use an independent age-matched or age-adjusted cohort and a fully batch-balanced design in which BrC and comparator samples are randomized across the same dates and plates, with blinded sample codes, shared pooled-QC material, prespecified preprocessing, and a locked feature/model pipeline. If discriminatory m/z features remain stable under those conditions, orthogonal molecular identification should then be pursued. These steps are required before cancer-specific diagnostic or biomarker claims are appropriate.

From a clinical perspective, the proposed approach should remain positioned as an adjunctive serum profiling strategy rather than as a replacement for mammography, ultrasonography, magnetic resonance imaging, or biopsy. A future role, if validated, could be as a low-complexity triage or complementary assessment tool. Prospective multicenter studies with clinically relevant comparator groups, including benign breast disease, inflammatory conditions, and other malignancies, will be necessary to establish disease specificity and real-world utility.

## 5. Conclusions

Workflow A provides preliminary proof-of-concept evidence that direct-serum MALDI-TOF/MS spectra can discriminate between the studied BrC and control groups (cross-validated AUC 0.932), but the substantial age imbalance means that cancer specificity cannot be established from this dataset. Workflow B showed high apparent internal discrimination after ACN-assisted preparation, but severe batch/date/source structure prevents attribution of that performance to malignancy.

Accordingly, Workflow B is interpreted as an exploratory analytical stress test rather than independent biological replication or validation. Its unidentified m/z bins are hypothesis-generating spectral features, not validated biomarkers. No claim is made that the two workflows detect the same molecular species.

The critical next step is an independent, age-matched or age-adjusted, same-day/same-plate randomized and blinded validation with shared pooled QC, complete acquisition metadata, sample-level replicate grouping, and a locked analysis pipeline. Orthogonal molecular identification should follow only for features that remain reproducible under those conditions.

## Figures and Tables

**Table 1:** Overview of the two serum MALDI-TOF/MS workflows evaluated in this study.

Characteristic	Workflow A: Direct Serum Profiling	Workflow B: ACN-Assisted Profiling
Biological/sample units	60 (30 BrC, 30 controls)	40 (20 BrC, 20 negative/comparator)
Technical measurements	Quadruplicate spectra averaged before modeling	Approximately four technical spots/sample; 160 spectra total
Sample preparation	1:5 serum dilution; direct matrix mixing	100% ACN 20 µL + serum 10 µL (2:1, *v*/*v*); centrifugation; supernatant
Nominal m/z range	2000–30,000	1000–19,500
Primary evaluation	Sample-level nested stratified 10-fold CV	Repeated sample-level five-fold CV (10 repeats) with training-fold-only feature selection
Primary reported discrimination	AUC 0.932	Apparent internal discrimination: mean AUC 0.978 ± 0.007; balanced accuracy 0.968
Interpretive role	Primary direct-workflow proof-of-concept	Exploratory analytical stress test with batch/source diagnostics

BrC, breast cancer; ACN, acetonitrile; AUC, area under the receiver operating characteristic curve; CV, cross-validation; MALDI-TOF/MS, matrix-assisted laser desorption/ionization time-of-flight mass spectrometry.

**Table 2:** Demographic and clinical characteristics of the direct-profiling subset.

Characteristic	BrC	Controls
No. of patients	30	30
Female	30	30
Age, mean (SD), years	52 (7.7)	45 (5.8)
Age range, years	34–64	36–56
Stage II/III	20/10	Not applicable
BI-RADS	Not applicable	I–III

BI-RADS, Breast Imaging Reporting and Data System; SD, standard deviation; BrC, breast cancer.

**Table 3:** Composition and batch structure of the ACN-assisted experimental set.

Group/Acquisition Structure	No. of Sample Groups	Age Information
BrC, 7 August 2026 P1	10	Included within BrC mean 48.25 y (31–66)
BrC, 7 August 2026 P2	10	Included within BrC mean 48.25 y (31–66)
Non-Br, 7 August 2026 P3	5	Included within Non-Br mean 50.8 y (40–62)
Lampire, 7 August 2026 P3	5	Unknown
Non-Br, 12 August 2026 P2	10	Included within Non-Br mean 50.8 y (40–62)
Total	40	20 BrC + 20 Negative/comparator

ACN, acetonitrile; BrC, breast cancer; Non-Br, non-breast comparator as labeled in the experimental plan.

**Table 4:** Summary of discrimination and batch-diagnostic results across the two workflows.

Analysis	Result	Interpretation
Direct workflow, *n* = 60	AUC 0.932	Primary direct-workflow proof-of-concept
ACN workflow, repeated five-fold CV ×10	AUC 0.978 ± 0.007	Apparent internal discrimination; confounded by batch/source structure
ACN workflow, mean balanced accuracy	0.968	Internal repeated-CV estimate; not disease-specific validation
ACN repeated OOF probability aggregation	AUC 0.995	Secondary descriptive estimate; not external validation
Negative/comparator batch/date classification	AUC 0.970	Strong batch-associated signal
Same-day BrC P1 vs. P2 plate classification	AUC 0.636	Smaller plate signal within same-day BrC samples
Cross-batch transfer	AUC 1.00/0.64	Direction-dependent transfer; severe instability and confounding

OOF, out-of-fold; BrC, breast cancer; ACN, acetonitrile; AUC, area under the receiver operating characteristic curve; CV, cross-validation.

## Data Availability

The direct-profiling dataset is publicly available in the Zenodo repository at https://doi.org/10.5281/zenodo.15803602 (accessed on 16 October 2026). The de-identified sample-level dataset, batch metadata, and analysis scripts for the ACN-assisted experiment are available from the corresponding authors upon reasonable request.

## Supplementary Materials

The following supporting information can be downloaded at: https://www.mdpi.com/article/doi/s1, Figure S1, TIC-normalized intensity comparisons between controls (n = 30) and breast cancer samples (n = 30) across m/z values in the direct serum profiling subset; Figure S2, Heatmap showing differential patterns of statistically significant m/z peaks between 30 controls and 30 BrC samples in the direct serum profiling subset; Figure S3, Waterfall plot showing spectral pattern differences among statistically significant m/z peaks between 30 controls and 30 breast cancer samples in the direct serum profiling subset; Figure S4, Representative direct-workflow MALDI-TOF/MS comparisons of six highlighted signals (m/z ~2101, 2550, 3258, 4150, 4207, and 5001) between controls (n = 30) and breast cancer samples (n = 30); Figure S5, Heatmap of the top 30 m/z features showing strong group-associated patterns in the ACN-assisted experimental set; Figure S6, Exploratory ACN candidate m/z analyses; Figure S7, Within-sample technical-replicate correlation in the ACN-assisted experiment; Table S1, Repeated 5-fold cross-validation results for the ACN-assisted experiment; Table S2, Exploratory unidentified discriminatory m/z bins in the ACN-assisted experiment; Table S3, Batch and transfer diagnostics in the ACN-assisted experiment; Table S4, Descriptive cross-workflow proximity check between highlighted direct-workflow signals and prioritized ACN bins.

## Abbreviations

The following abbreviations are used in this manuscript:

ACN	Acetonitrile
AUC	Area under the receiver operating characteristic curve
BI-RADS	Breast Imaging Reporting and Data System
BrC	Breast cancer
CV	Cross-validation
MALDI-TOF/MS	Matrix-assisted laser desorption/ionization time-of-flight mass spectrometry
Non-Br	Non-breast comparator label used in the experimental plan
QC	Quality control
PCA	Principal component analysis
TIC	Total ion current
