# Supplementary material for: MALDI-TOF/MS Serum Spectral Profiling for Breast Cancer Discrimination Using Direct and ACN-Assisted Workflows: A Retrospective Proof-of-Concept Study
Source: Diagnostics (Basel). 2026 Oct 27;16(21):3520. doi: 10.3390/diagnostics16213520 (PMC13761583; doi:10.3390/diagnostics16213520)
Supplement: Supplementary file 1 [file diagnostics-16-03520-s001.zip › diagnostics-4565201-supplementary.pdf]

diagnostics

Article

MALDI-TOF/MS Serum Spectral Profiling for Breast Cancer Discrimination Using Direct and ACN-Assisted Workflows: A Retrospective Proof-of-Concept Study

Donggun Seo[1,†], Soojeong Choi[2,†], Hyeongjin Kweon[1], Sae Byul Lee[3,*], Dong-Chan Kim[1,*]

1 R&D Center, ASTA Inc., 767, Sinsu-ro, Yongin-si, Gyeonggi-do, 16827, Republic of Korea; jenokin@nate.com
2 Department of Surgery, Seoul Medical Center, 156, Sinnae-ro, Jungnang-gu, Seoul, 02053, Republic of Korea
3 Division of Breast Surgery, Department of Surgery, Asan Medical Center, University of Ulsan College of Medicine, 88, Olympic-ro 43-gil, Songpa-gu, Seoul, 05505, Republic of Korea; newstar153@hanmail.net
† These authors contributed equally to this work and share first authorship
* Correspondence: newstar153@hanmail.net; Tel.: +82-10-7209-4620; Fax: +82-2-3010-6863 (S.B.L.); jenokin@nate.com; Tel.: +82-10-4527-3736; Fax: +82-31-624-5162 (D.-C.K.)

diagnostics

MDPI

Supplementary Materials

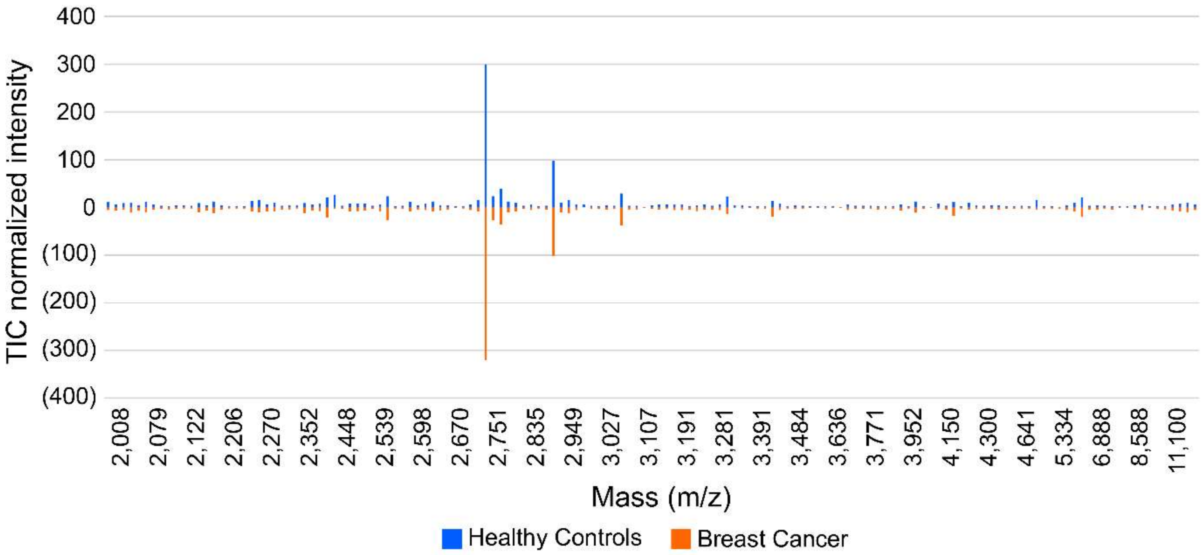

Figure S1. TIC-normalized intensity comparisons between controls (n = 30) and breast cancer samples (n = 30) across m/z values in the direct serum profiling subset.

TIC, total ion current.

diagnostics

MDPI

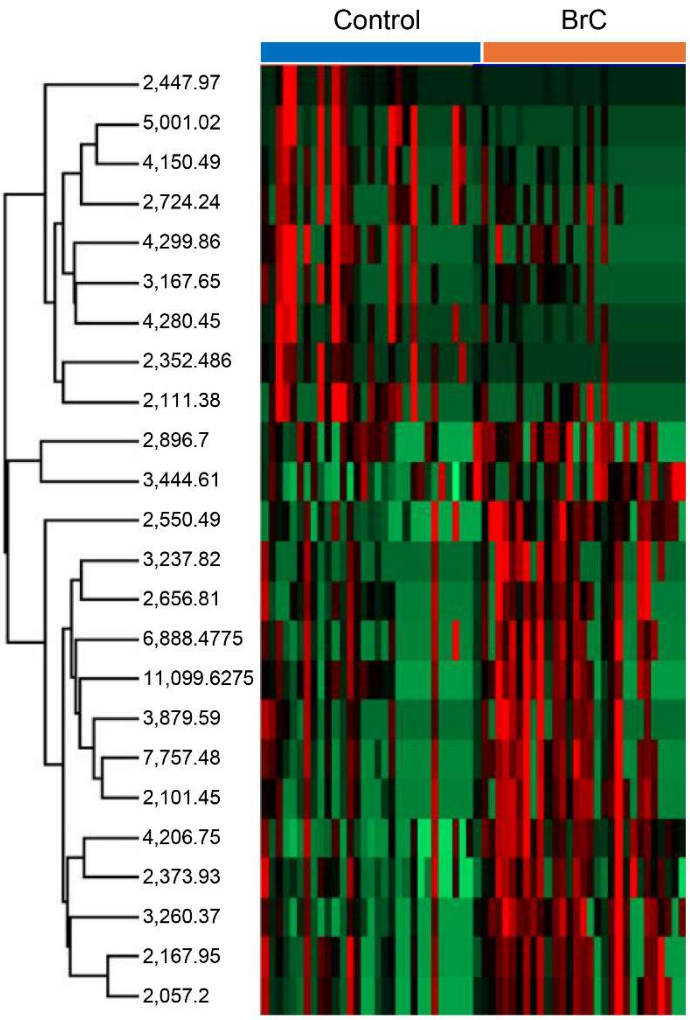

Figure S2. Heatmap showing differential patterns of statistically significant m/z peaks between 30 controls and 30 BrC samples in the direct serum profiling subset.

BrC, breast cancer.

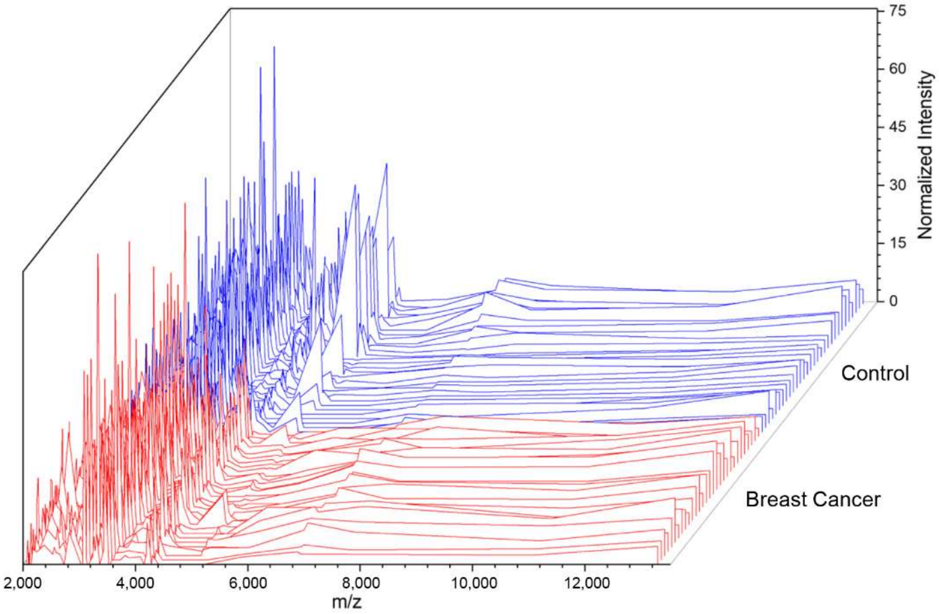

Figure S3. Waterfall plot showing spectral pattern differences among statistically significant m/z peaks between 30 controls and 30 breast cancer samples in the direct serum profiling subset.

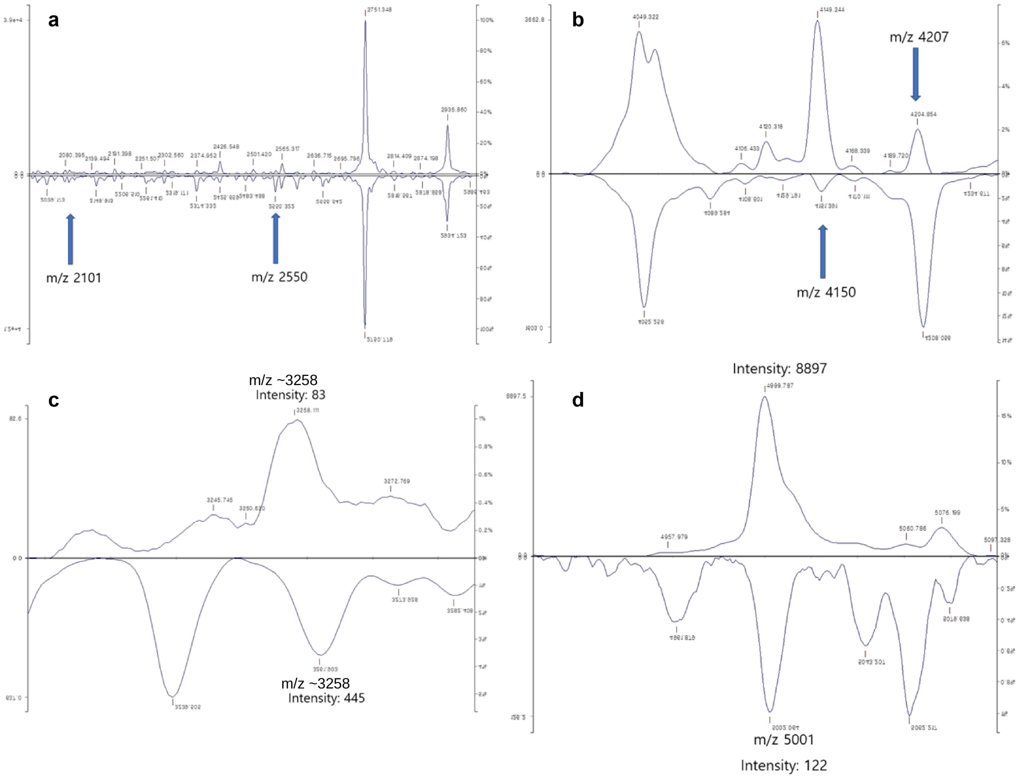

Figure S4. Representative direct-workflow MALDI-TOF/MS comparisons of six highlighted signals (m/z ~2101, 2550, 3258, 4150, 4207, and 5001) between controls (n = 30) and breast cancer samples (n = 30). Panel (c) is labeled as the ~m/z 3258 region. These signals are descriptive and have not been molecularly identified.

MALDI-TOF/MS, matrix-assisted laser desorption/ionization time-of-flight mass spectrometry

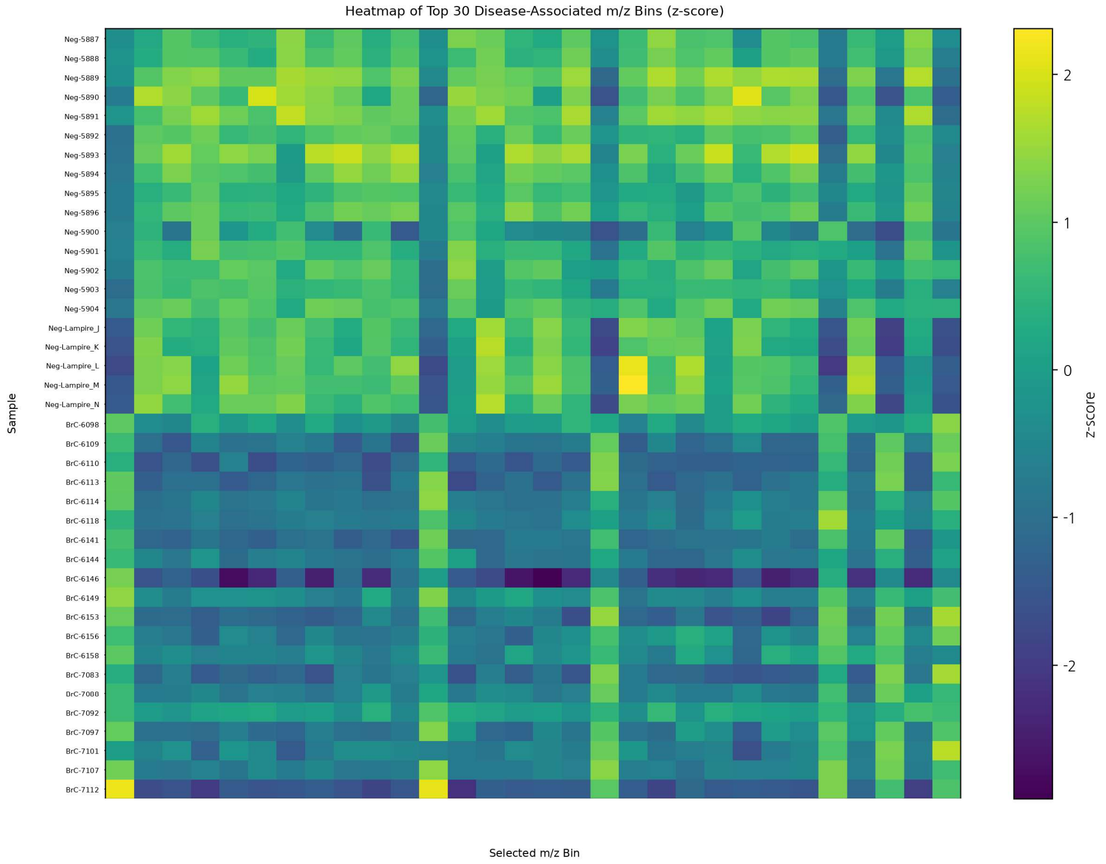

Figure S5. Heatmap of the top 30 m/z features showing strong group-associated patterns in the ACN-assisted experimental set. Because disease status, specimen source, measurement date, and plate were not independently balanced, this heatmap is not interpreted as evidence of disease-specific biology and should be read together with the batch/source diagnostics in the main manuscript.

ACN, acetonitrile

a

b

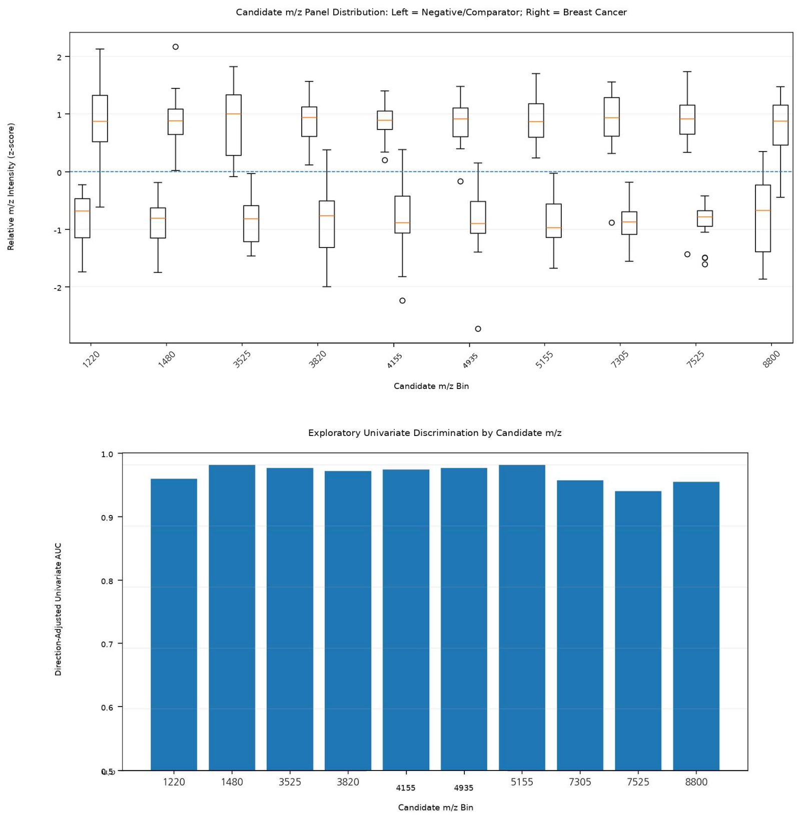

Figure S6. Exploratory ACN candidate m/z analyses. (a) Distribution of the 10 prioritized unidentified discriminatory m/z bins in breast cancer and negative/comparator samples. (b) Direction-adjusted univariate AUC values for the same bins. These values were calculated within the confounded discovery dataset, are not independent validation results, and do not establish molecular identity or cross-workflow molecular concordance.
ACN, acetonitrile; AUC, area under the receiver operating characteristic curve

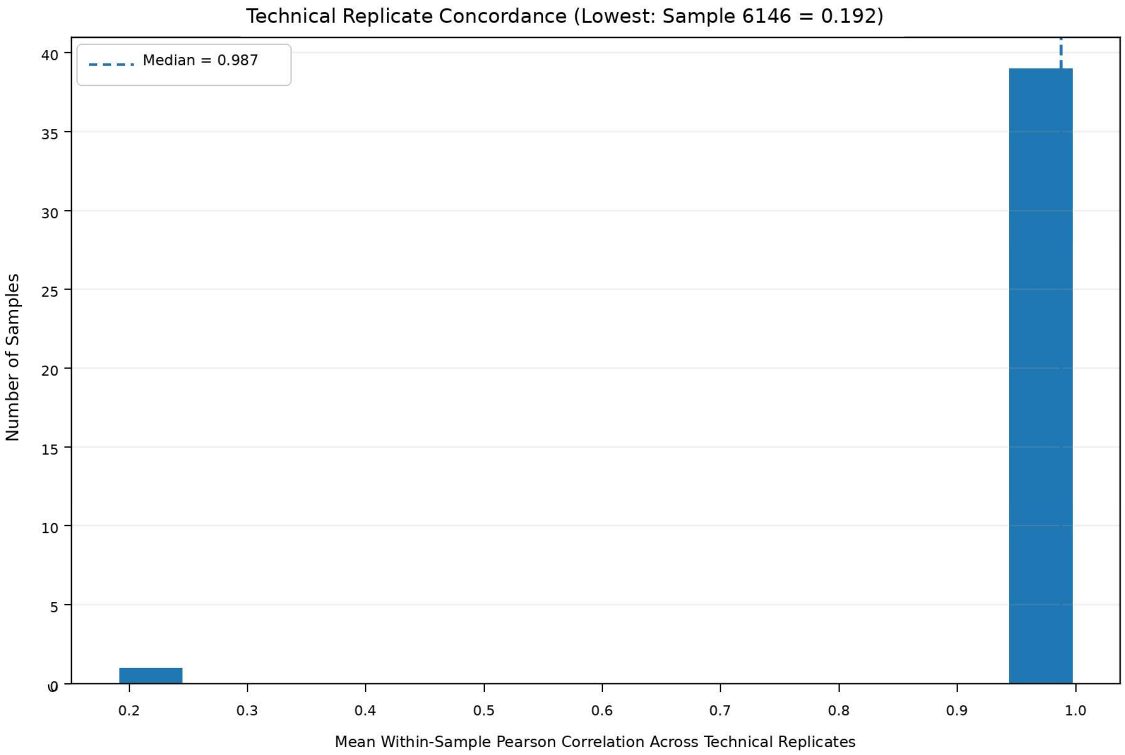

Figure S7. Within-sample technical-replicate correlation in the ACN-assisted experiment. Most sample groups showed high replicate correlation; breast cancer sample 6146 showed a marked technical-replicate anomaly and was treated as a QC concern in sensitivity analysis.

ACN, acetonitrile; QC, quality control

Table S1. Repeated 5-fold cross-validation results for the ACN-assisted experiment

Repeat	AUC	Balanced accuracy	Sensitivity	Specificity
1	0.9725	0.975	1.00	0.95
2	0.9850	0.925	0.90	0.95
3	0.9800	0.975	1.00	0.95
4	0.9775	0.975	1.00	0.95
5	0.9900	0.950	0.95	0.95
6	0.9775	0.975	1.00	0.95
7	0.9800	0.975	1.00	0.95
8	0.9700	0.975	1.00	0.95
9	0.9775	0.975	1.00	0.95
10	0.9675	0.975	1.00	0.95

Mean AUC = 0.978 ± 0.007; mean balanced accuracy = 0.968. Values are derived from repeated internal cross-validation of the same 40 sample groups and do not represent external validation.

AUC, area under the receiver operating characteristic curve; ACN, acetonitrile.

Table S2. Exploratory unidentified discriminatory m/z bins in the ACN-assisted experiment

m/z	Direction in BrC	Cohen's d	Direction-adjusted univariate AUC	CV selection frequency	q-value
5155	Decreased	−4.27	1.000	82%	2.43×10^{-5}
1480	Increased	3.81	1.000	80%	2.43×10^{-5}
7305	Decreased	−3.68	0.975	78%	2.43×10^{-5}
7525	Decreased	−3.31	0.958	60%	2.91×10^{-5}
3525	Decreased	−3.40	0.995	56%	2.43×10^{-5}
4935	Decreased	−3.31	0.995	48%	2.43×10^{-5}
3820	Decreased	−3.23	0.990	44%	2.43×10^{-5}
4155	Decreased	−3.26	0.993	44%	2.43×10^{-5}
1220	Increased	2.93	0.978	34%	2.43×10^{-5}
8800	Increased	2.64	0.973	26%	2.43×10^{-5}

Direction-adjusted univariate AUC values reflect separation within the discovery dataset only. Candidate values are 5-m/z bins, have not been molecularly identified, and should not be interpreted as validated biomarkers or as evidence of the same molecular species detected in Workflow A.

ACN, acetonitrile; BrC, breast cancer; CV, cross-validation; AUC, area under the receiver operating characteristic curve

Table S3. Batch and transfer diagnostics in the ACN-assisted experiment

Diagnostic analysis	AUC	Interpretation
Negative/comparator: 2026-08-07 P3 vs 2026-08-12 P2	0.970	Strong date/batch signal
Breast cancer, same day: P1 vs P2	0.636	Relatively smaller plate signal
Cross-batch transfer, direction 1	1.00	High performance in one transfer direction
Cross-batch transfer, reverse direction	0.64	Substantial instability
Reverse direction excluding Lampire	0.74	Partial improvement; instability remains

ACN, acetonitrile; AUC, area under the receiver operating characteristic curve. These diagnostics show substantial date/plate/source dependence; therefore, the ACN workflow is interpreted as an exploratory analytical stress test rather than cancer-specific validation.

Table S4. Descriptive cross-workflow proximity check between highlighted direct-workflow signals and prioritized ACN bins

Workflow A highlighted signal (m/z)	ACN bin within ±5 Da	Δm/z (Da)	Interpretation
2101	—	—	No ACN bin within ±5 Da
2550	—	—	No ACN bin within ±5 Da
~3258	—	—	No ACN bin within ±5 Da
4150	4155	+5	Near-coincident descriptive pair only
4207	—	—	No ACN bin within ±5 Da
5001	—	—	No ACN bin within ±5 Da

The 10 prioritized ACN bins were m/z 1220, 1480, 3525, 3820, 4155, 4935, 5155, 7305, 7525, and 8800. ACN bins 1220 and 1480 lie below the nominal Workflow A acquisition range (m/z 2000–30,000). The 4150/4155 proximity (Δ ≈ 5 Da; ≈0.12%) is compatible with the descriptive tolerance imposed by linear-mode peak localization and 5-m/z binning, but it is not evidence of a shared molecular species or cross-workflow biological replication.
